# Going slippery for a robust triboelectric nanogenerator

**DOI:** 10.1093/nsr/nwz130

**Published:** 2019-09-02

**Authors:** Wenluan Zhang, Qiangqiang Sun, Xu Deng

**Affiliations:** 1 School of Automation Engineering, University of Electronic Science and Technology of China, China; 2 Institute of Fundamental and Frontier Sciences, University of Electronic Science and Technology of China, China

Nowadays, 17% global electricity and 70% renewable electricity are from hydropower. Despite its abundance and sustainability, converting hydropower to electricity always needs turbines and magnets to make use of electromagnetic induction [1]. Unlike electromagnetic induction, direct contact between materials can also generate electric charges. This contact electrification or triboelectricity phenomenon was discovered more than 2000 years ago, but its application in energy-related fields has not been greatly explored until recently, when Wang *et al.* developed a triboelectric nanogenerator (TENG) to harvest the kinetic energy from water droplets to produce electricity, which allows us to access hydropower in a wide range of working environments [2].

**Figure 1.**
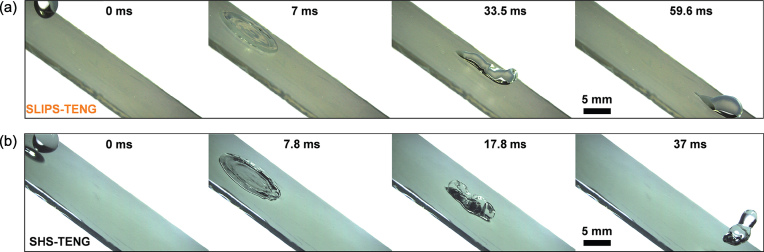
(a) A droplet falling on SLIPS-TENG is in intimate contact with the lubricant-infused interface and slides out of view. (b) In contrast, the droplet bounces off SHS-TENG in a much shorter period. This intimate and large contact area between the droplet and SLIPS is believed to play a key role in this robust water-TENG. Reproduced from [3].

Facing these challenges, writing in *National Science Review*, Xu *et al.* proposed a slippery lubricant-impregnated porous surface (SLIPS) for robust TENGs [3]. They infused perfluorinated liquid into porous PTFE membrane, then deposited this SLIPS on a patterned indium tin oxide electrode for collecting electric current induced by falling water droplets. The authors claimed that this *Nepenthes*-pitcher-inspired strategy gives the device multiple advantages [4], because of the replacement of a solid/air/liquid interface with a liquid/liquid interface. Meanwhile, in freezing temperatures, the lubricant can hinder the formation of ice crystals. Additionally, owing to the flowability of the lubricant, the surface can recover after a scratch without weakening the electricity output performance. The voltage and current generated from SLIPS-TENG show similar intensity compared with those from SHS-TENG. The authors attribute this result to the large area and intimate prolonged contact between the water droplet and the lubricant as shown in Fig. 1. They also found a critical thickness of the lubricant film, below which the amounts of charges from the droplet/lubricant and droplet/PTFE interfaces are almost identical. So in this case, does the electricity originate from the water/PTFE interface, the water/lubricant interface, or a type of combined effect? The authors compared the charges generated from an impacting water droplet on various interfaces to claim that water/PTFE is still responsible for the observed triboelectricity. This is mainly because the charges from droplets falling on SLIPS-TENG (3 nC/g) and water/SHS (3.5 nC/g) are two orders larger than those from water/lubricant and lubricant/PTFE interfaces. However, unlike previously described droplet charges from solid/liquid and liquid/liquid interfaces [5,6], the role played by SLIPS, involving both solid and liquid concurrently, in the fundamental mechanism of this interesting phenomenon remains elusive.

At this stage, SLIPS-TENG is still not better than SHS-TENG in terms of electricity output and the design is ripe for optimization. Furthermore, the mechanism of charge transparency still needs to be investigated in great detail. Nevertheless, this is the first work combining SLIPS and TENG, representing the latest effort towards a robust TENG. This work will encourage the search for high-performance TENGs, and might eventually enable their application in real life.

## References

[1] Siria A , Bocquet ML, Bocquet L. Nat Rev Chem 2017; 1: 0091.

[2] Lin Z-H , Cheng G, Lee S et al. Adv Mater 2014; 26: 4690–6. 24830874 10.1002/adma.201400373

[3] Xu W , Zhou X, Hao C et al. Natl Sci Rev 2019; 6: 540–50. 10.1093/nsr/nwz025 PMC8291521 34691903

[4] Wong TS , Kang SH, Tang SK et al. Nature 2011; 477: 443–7. 21938066 10.1038/nature10447

[5] Wang ZL , Wang AC. Mater Today 2019; 30: 34–51.

[6] Nie J , Wang Z, Ren Z et al. Nat Commun 2019; 10: 2264. 31118419 10.1038/s41467-019-10232-x PMC6531479

